# Homogeneous Free-Standing Nanostructures from Bulk Diamond over Millimeter Scales for Quantum Technologies

**DOI:** 10.1021/acs.nanolett.5c03083

**Published:** 2025-09-26

**Authors:** Andrea Corazza, Silvia Ruffieux, Yuchun Zhu, Claudio A. Jaramillo Concha, Yannik Fontana, Christophe Galland, Richard J. Warburton, Patrick Maletinsky

**Affiliations:** † Department of Physics, 27209 University of Basel, CH-4056 Basel, Switzerland; ‡ Institute of Physics and Center for Quantum Science and Engineering, 27218 Ecole Polytechnique Fédérale de Lausanne (EPFL), 1015 Lausanne, Switzerland

**Keywords:** Diamond nanostructures, Diamond photonics, Quantum communication, Quantum sensing, Photonic crystal cavities, Optical lithography

## Abstract

Quantum devices based on optically addressable spin qubits in diamond are promising platforms for quantum technologies such as quantum sensing and communication. Nano- and microstructuring of the diamond crystal is essential to enhance device performance, yet fabrication remains challenging and often involves trade-offs in surface quality, aspect ratio, device size, and uniformity. We tackle this hurdle with an approach producing millimeter-scale, thin (down to 70 nm), and highly parallel (< 0.35 nm/μm) membranes from single-crystal diamond. The membranes remain contamination free and possess atomically smooth surfaces (R_q_ <  200 pm) as required by state-of-the-art quantum applications. We demonstrate the benefits and versatility of our method by fabricating large fields of free-standing and homogeneous photonic nano- and microstructures. Leveraging a refined photolithography-based strategy, our method offers enhanced scalability and produces robust structures suitable for direct use, while remaining compatible with heterogeneous integration through pick-and-place transfer techniques.

## Graphical abstract

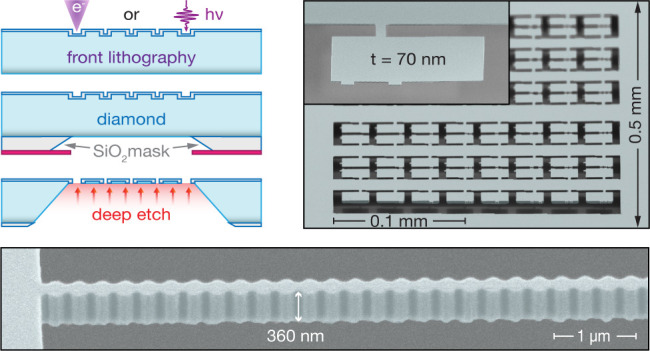

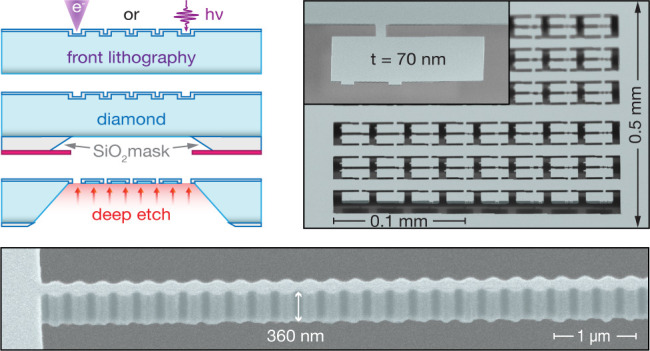

Diamond’s unparalleled properties make it an enticing choice for a plethora of applications, including low-loss microelectro-mechanical systems,^1–3^ high-power electronics and optics,^4–7^ as well as quantum photonics, phononics, and sensing.^8–10^ Notably, diamond can incorporate a variety of optically active point defects (color centers) that also exhibit coherent ground-state spins.^11,12^ The intrinsic spin-photon interface provided by color centers in single-crystal diamond has enabled multiple breakthroughs pertaining to quantum registers and networks.^13–17^ Additionally, their spin can serve as powerful sensors either as ensembles^18–20^ or at the single center level.^21–23^ At the core of most of these achievements is the ability to process diamond at the micro- and nanoscale. Novel fabrication methods enabled advanced diamond quantum devices such as micrometer-thin membranes for heterogeneous photonics,^24–28^ all diamond scanning probe sensors,^29–32^ mechanical resonators,^1,33–37^ and monolithic nanophotonic waveguides and photonic crystal (PhC) cavities.^38–41^ However, in several aspects, fabrication hurdles continue to limit the performance and scalability of these devices. Specifically, obtaining high-quality, extended, and homogeneous arrays of devices with arbitrary geometries while maintaining a high yield and integration potential remains a stumbling block. Additionally, for color center-based devices, magnetic and charge fluctuators located on processed surfaces lead to a degradation of the spin and optical coherence of the color centers, emphasizing the need for well-terminated, atomically smooth diamond surfaces.^42,43^

The fabrication of released, free-standing devices is a key aspect of this challenge, and several methods have emerged to address it.^44–46^ Angled-etching^47–49^ and quasi-isotropic etching^50–52^ rely on under-etching of structures fabricated on bulk diamond surfaces and work best for low-dimensional, thin structures or devices supported by a pedestal. The downsides of either are the tendency to produce suboptimal surface quality (increased roughness) and the inability to obtain arbitrarily flat back surfaces, compromising the device performance.^41^ A different strategy to produce diamond membranes with thicknesses matching the device requirement proceeds via a “smart-cut” process combined with diamond epitaxy.^53,54^ This method yields thin membranes, typically ranging from tens to a few hundred nanometers,^55,56^ with higher surface quality compared to the underetching methods. However, the smart-cut approach is complex and resource intensive, and the membranes are completely disconnected from the bulk diamond and therefore need to be rebonded to a carrier.^57^

A more straightforward procedure for fabricating free-standing devices involves deep etching of a bulk diamond over an area defined by macroscopic shadow masks. By this approach, membranes can be created on millimeter scales, with low-roughness surfaces and a diamond quality limited only by the starting material.^30,58^ Yet, obtaining submicrometer thicknesses and low gradients across the entire etched membrane is highly challenging because of geometry-induced plasma enhancement effects^59,60^ that result in micrometer-deep trench build-ups along the membrane’s perimeter (Figure 1A). Consequently, the minimum achievable thickness of the free-standing structures is limited. Reduction of the plasma flux near the sidewalls can be achieved using a shadow mask with ∼30° sidewalls (wider opening facing the diamond, Figure 1B); however, this method still produces significant thickness gradients that are dependent on the mask geometry.^34,60^ Additionally, achieving accurate mask alignment and reliable mask fixation during the deep etch process remains challenging.^58^

**Figure 1:**
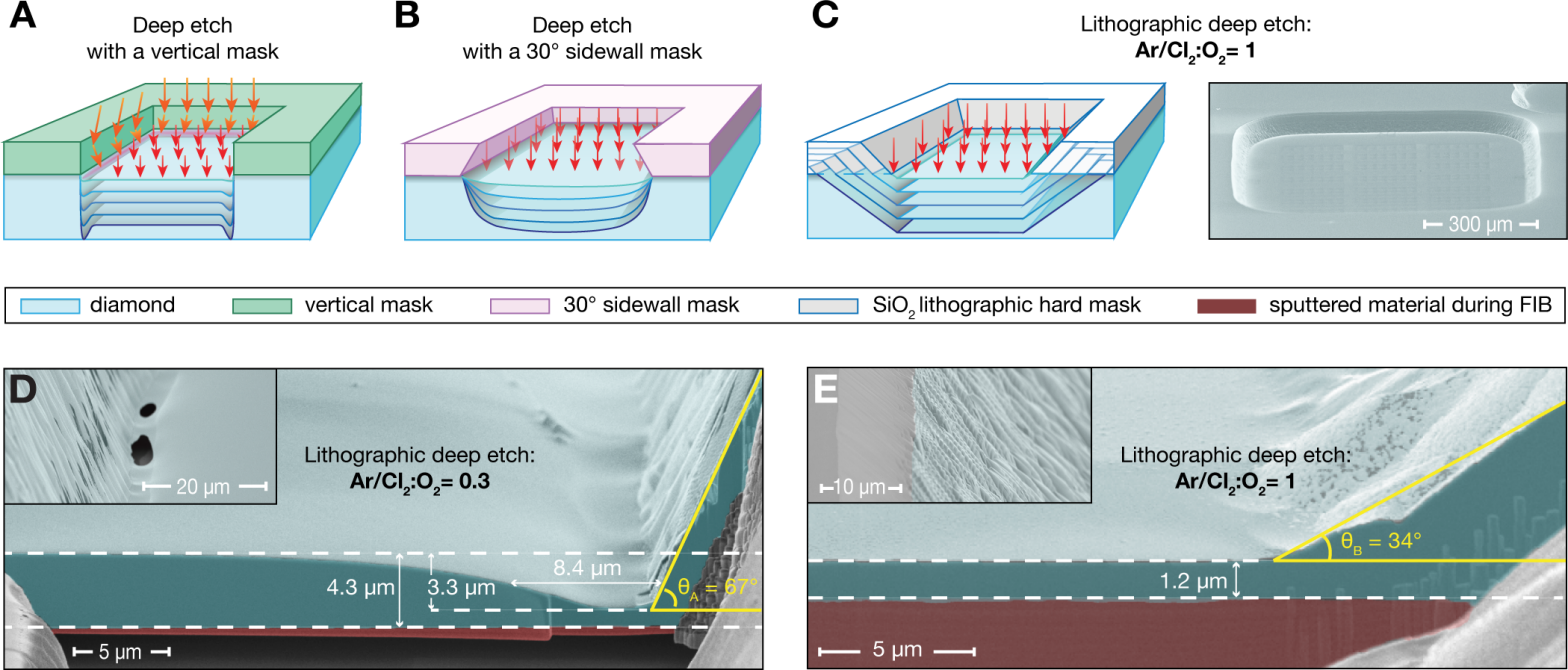
Diamond membrane processing. (A) A vertical hard mask (green) and vertical diamond (blue) sidewall confine the etching plasma (orange arrows) at the edges of the etching pit, leading to the formation of trenches. (B) A mask with ∼30° sidewalls (pink) decreases the etching plasma density (faded red arrows) at the edges of the etching pit, mitigating the formation of trenches but introducing thickness gradients in the membrane. (C) Left: Evolution of the etching pit profile using an Ar/Cl_2_:O_2_ ratio of 1. The angled sidewall of the pit, created by etching the SiO_2_ mask during the Ar/Cl_2_ steps, mitigates plasma confinement and prevents trench formation. Right: SEM micrograph (taken at 70° viewing angle) of a ∼45 μm deep etching pit. The resulting diamond membrane has an area of ∼760 × 760 μm^2^ and a thickness of 1.2 μm. (D) SEM micrograph (same imaging angle) of the membrane thickness profile near the etching pit sidewall for an Ar/Cl_2_:O_2_ ratio of 0.3. The trench is 3.3 μm deep and 8.4 μm wide at half-depth. Inset: Perforation of the membrane due to trenching, compromising its mechanical stability. (E) Same as (D), but for an Ar/Cl_2_:O_2_ ratio of 1. No trenches are visible. Inset: Profile of the etching pit sidewall and of the SiO_2_ mask during etching. Terraces form due to different selectivity of the Ar/Cl_2_ and O_2_ plasma.

In addition to the ability to create thin structures, precise and efficient micro- and nanoscale patterning of diamond surfaces is essential for fabricating diamond-based quantum devices. Diamond’s hardness and chemical inertness demand mask materials with high chemical selectivity to transfer accurately the designed patterns.^44^ Techniques such as electron beam lithography (EBL)^47,51^ and focused ion beam (FIB) milling^61,62^ have been successfully used to fabricate photonic crystal nanocavities and waveguides. However, while achieving high accuracy and resolution, these methods are resource intensive and inefficient for large-scale patterning of micrometer-sized structures, such as scanning probe sensors^30^ and platelets for heterogeneous photonic devices.^24^ Additionally, ion beam irradiation risks damaging the diamond surface and embedded color centers. Optical lithography is a fast and scalable alternative, and it has already been used to pattern diamond structures.^31,63,64^ However, further development is needed to reproducibly reach the submicrometer resolution required to break out free-standing quantum devices by pick-and-place techniques.

In this work, we present first an improved fabrication strategy, the “lithographic deep etch” (LDE), to produce large-scale free-standing thin membranes. The LDE 1) eliminates problems with trench formation and thickness inhomogeneities, 2) enables high-accuracy positioning of the area to be deep-etched, and 3) allows us to define multiple etching windows of arbitrary shapes on one and the same diamond. To demonstrate the potential of this method for quantum photonics, we pattern the front surface of bulk diamonds with micro- and nanostructures (diamond platelets for heterogeneous integration and PhC cavities, respectively) that are subsequently released from the back by a deep etch. The free-standing structures we obtain have a submicrometer thickness (down to 70 nm) with a residual gradient limited only by the initial wedge of the diamond plate. They are compatible with pick-and-place transfer techniques while remaining supported by a frame robust enough for manual manipulation. In a second part, we present a fast, simple, and scalable approach using optical lithography combined with an active feedback focusing system to define the front pattern of microstructures into diamond. To demonstrate its capability, we fabricate arrays of diamond platelets for heterogeneous integration and cantilevers for scanning-probe sensors, achieving submicrometer resolution.

The LDE process for creating large-scale free-standing membranes from a bulk diamond (∼50 μm) relies on creating a thick SiO_2_ lithographic mask with outward-slanted sidewalls that retract as the deep etch progresses (Figure 1C). Our inductively coupled plasma reactive ion etching (ICP-RIE) recipe relies on alternating Ar/Cl_2_ and O_2_ plasmas.^29^ The former mostly leads to a smoothening of the diamond surface, while the latter produces a fast, anisotropic etch of the diamond. Crucially, Ar/Cl_2_ also etches aggressively SiO_2_. Thus, tuning the etching time ratio of the plasmas allows for controlled lateral retraction of the SiO_2_ mask. As a result, the angle of the etching pit’s sidewalls can be tuned, preventing plasma confinement and the formation of trenches. The mechanism that avoids the trench formation during the LDE is shown in Figure 1C, together with a typical 45 μm deep etching pit resulting from our process, here with an area of 760 × 760 μm^2^. The diamond membrane is 1.2 μm thick. To characterize how the membrane thickness profile close to the diamond sidewall evolves as a function of the Ar/Cl_2_:O_2_ content, we mill an inspection slot in the proximity of the etching pit sidewall via a focused ion beam (FIB) to access the trench profiles. In Figure 1D, where a diamond pit etched with an Ar/Cl_2_:O_2_ ratio of 0.3 is shown, a trench with a depth of 3.3 μm and a width of 8.4 μm at half its depth is measured. The angle of the diamond etching pit sidewall from the sample plane is 67°, showing that the plasma confinement, although mitigated, is still present and induces higher etching rates along the membrane perimeter and eventually leads to perforation (see inset of Figure 1D). Increasing the Ar/Cl_2_:O_2_ ratio to one decreases the diamond etching pit sidewall angle close to the membrane to 34° (Figure 1E), while the sidewall has an average angle of ∼45° (inset Figure 1E). At this sidewall angle, the reflected ions at grazing angles no longer reach the diamond membrane but hit the sidewall such that no trench build-up is observed. In the inset of Figure 1E the diamond terraces, created by alternating the two plasmas, as well as the SiO_2_ lithographic mask are visible.

To integrate patterns needed for quantum devices into the diamond membrane, two approaches can be employed. In the first approach, the design (“front lithography”) is patterned prior to membrane release, while in the second, patterning is performed after the creation of a thin diamond membrane. For the fabrication of submicrometer free-standing diamond nanostructures, we adopt the first approach to avoid potential damage to the fragile membrane during lithography. The fabrication process for the LDE following the front pattern definition is illustrated in Figure 2A. First, we use plasma-enhanced chemical vapor deposition (PECVD) to deposit a layer of SiO_2_ (50–100 nm) to protect the front design, flip the sample, and deposit a thick (10–22 μm) layer of SiO_2_ as hard mask for the deep etch. Second, optical lithography is performed to define the etching windows (see Supporting Information (SI)), and the design is transferred to the hard mask via a buffered oxide etchant (BOE) 10:1 etch. The isotropic wet etch guarantees outward-slanted sidewalls with angles much lower than ∼45° near the diamond surface. This enables the hard mask to retract in the third step, in which the diamond is etched with alternating Ar/Cl_2_ and O_2_ plasmas in an ICP-RIE reactor. Fourth, once the desired membrane thickness is achieved, the mask is removed with a BOE (10:1) etch.

**Figure 2:**
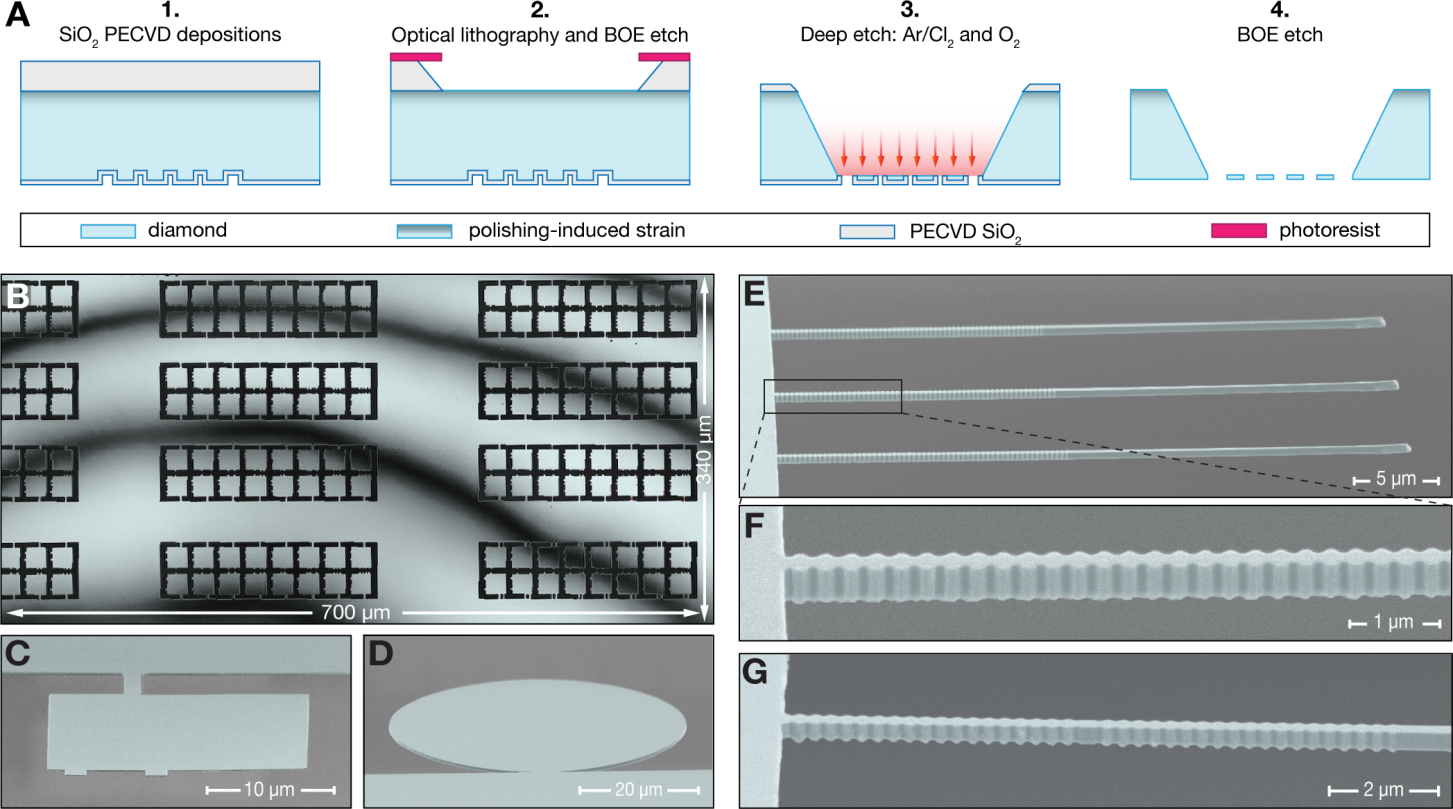
(A) Fabrication flow for the LDE process. (1) After the front pattern definition, the front surface is protected with a thin layer of SiO_2_ and the thick deep etch mask (10–22 μm of SiO_2_, light gray) is deposited on the backside. (2) Definition of the etching windows via optical lithography and a BOE etch to transfer the pattern to the hard mask. (3) Photoresist removal and ICP-RIE deep etch using Ar/Cl_2_ and O_2_ to release the nanostructures. (4) BOE etch to remove the hard mask. (B) Laser scanning confocal microscope (λ = 404 nm) image of a 700 × 340 μm^2^ region of the deep-etched side of a 2 × 1 mm^2^ free-standing membrane patterned with platelets. The membrane has a wedge less than 0.6 nm/μm along the highest thickness gradient direction. The thickness difference between fringes is 84 nm. (C) SEM micrograph of a single 70 nm thick free-standing platelet. (D) SEM micrograph of a 50 μm diameter disk with a thickness of 500 nm. (E) SEM micrograph of 360 nm thick free-standing tapered waveguides with distributed Bragg reflectors (DBR). (F) Zoom in on the DBR and anchor point to the holding structure of one of the waveguides shown in (E). (G) SEM micrograph of a 350 nm thick free-standing tapered PhC cavity. All SEM images were obtained at a 70° viewing angle. The front pattern definition in (B) was done by optical lithography, while EBL was used to pattern the structures in (C)–(G).

We characterize the thickness gradient of the free-standing membranes using a laser scanning confocal microscope (Keyence VK-X1100, λ = 404 nm). The measured wedge is consistent with that observed for the diamond plate before the LDE (see SI Figure S3) and is solely determined by the intrinsic thickness gradient introduced during laser slicing and polishing. Thus, unlike deep etching approaches based on bulk masks,^58,60,65,66^ the LDE process does not introduce any additional thickness inhomogeneities. We quantify the wedge within a 700 × 340 μm^2^ region of a free-standing membrane patterned with platelets in Figure 2B knowing that the thickness difference between two destructive interference fringes is λ/2*n*_*d*_ = 84 nm, where *n*_*d*_ is the refractive index of diamond. The entire membrane has a wedge below 0.6 nm/μm along the direction of the highest thickness gradient. By characterizing the thickness gradient of the diamond plate before patterning devices, owing to the lithographic nature of the LDE, one can exploit the intrinsic wedge for tuning the thickness of the quantum devices. Over a 1 mm^2^ region, we measured wedges down to 0.35 nm/μm along the highest thickness gradient direction (see SI Figure S3), which projected on the typical size of our Fabry–Pérot microcavity platelets,^24,27^ 20 × 20 μm^2^, leads to a maximal thickness difference of 7 nm.

The absence of a trench and the flexibility of the LDE process enable the fabrication of free-standing diamond structures with arbitrary thicknesses and size. As visible in Figure 2C, extended free-standing areas as thin as 70 nm (see SI Figure S4) can be realized, demonstrating this method as a highly competitive alternative to smart-cut techniques. Moreover, the LDE process enables the creation of flat structures connected to a supporting frame and with arbitrary in-plane aspect ratios, exemplified in Figure 2D—which are beyond the reach of conventional angled-etching or quasi-isotropic etching methods. To demonstrate the potential of this approach, we fabricate some of the most demanding structures required for diamond-based quantum technologies, where stringent control over morphology and roughness of the back surface is critical. Specifically, we realize two distinct types of free-standing nanostructures, each with a thickness of 350–360 nm: tapered waveguides with distributed Bragg reflectors (DBRs) and tapered photonic crystal (PhC) cavities. Both designs are optimized for a target wavelength of 1042 nm, corresponding to the nitrogen-vacancy (NV) center’s singlet transition, with an ideal thickness of 355 nm. The full length of three tapered waveguides with DBRs is presented in Figure 2E, while a magnified view highlighting the DBR structure and the anchor points to the holding framework is shown in Figure 2F. Additionally, Figure 2G displays an SEM image of the two DBRs forming a PhC cavity, further illustrating the precision and viability of our fabrication process.

We characterize the surface morphology of the deep-etched side of the diamond nanostructures by using atomic force microscopy (AFM). To facilitate this, free-standing diamond platelets, approximately 900 nm thick, were released and bonded to a silicon chip via van der Waals forces, with the deep-etched surface facing upward (see SI Sec. IV). The morphology and roughness of this surface are compared to those of the front surface, which only underwent a 4 μm stress-relief etch to remove crystal damage introduced by the polishing.^30^ It is important to note that the plasma chemistry used for the deep etch closely mimics that employed to achieve state-of-the-art stress-relieved diamond surfaces, utilizing alternating Ar/Cl_2_ and O_2_ plasmas (see SI Sec. I). Figure 3A presents an AFM scan of the front surface after stress relief, revealing a remarkably low surface roughness (R_*q*_) of 188 pm, even over an area of 10 × 10 μm^2^. Notably, there is no significant increase in R_*q*_ when comparing a 1 × 1 μm^2^ scan (187 pm) to a 10 × 10 μm^2^ scan (see SI Figure S1D). This long-range flatness over tens of square micrometers is attributed to the combination of the stress-relief etching process and an improved diamond polishing technique developed by Almax easyLab (Belgium), which already yields surfaces with a roughness of approximately 200 pm after polishing (see SI Figure S1B). The exceptional surface quality and flatness of the released diamond micro- and nanostructures hold promise for photonics and quantum device applications, as polishing-induced surface waviness is a major limiting factor in achieving high Q-factors in diamond-based Fabry–Pérot microcavities.^67^ Figure 3B shows a 10 × 10 μm^2^ AFM scan of the deep-etched surface, revealing a surface roughness of 342 pm. While this initially suggests an increase in roughness due to the deep etch, it is crucial to consider that the back surface, before deep etching, had undergone a different polishing process performed by Almax easyLab, resulting in a significantly higher initial roughness of 976 pm over a 10 × 10 μm^2^ area (see SI Figure S1A). To allow for a fair comparison, we applied our standard stress-relief etch to a diamond sample that underwent the same initial polishing as the deep-etched surface. The resulting R_*q*_ value of 587 pm indicates that the deep etch effectively smoothens the waviness induced by polishing, even more so than the stress-relief etch (see SI Figure S1C). Finally, it is worth noting that polishing-induced waviness becomes relevant only for surface areas larger than 1 × 1 μm^2^, as at that scale, R_*q*_ remains below 200 pm for all considered cases.

**Figure 3:**
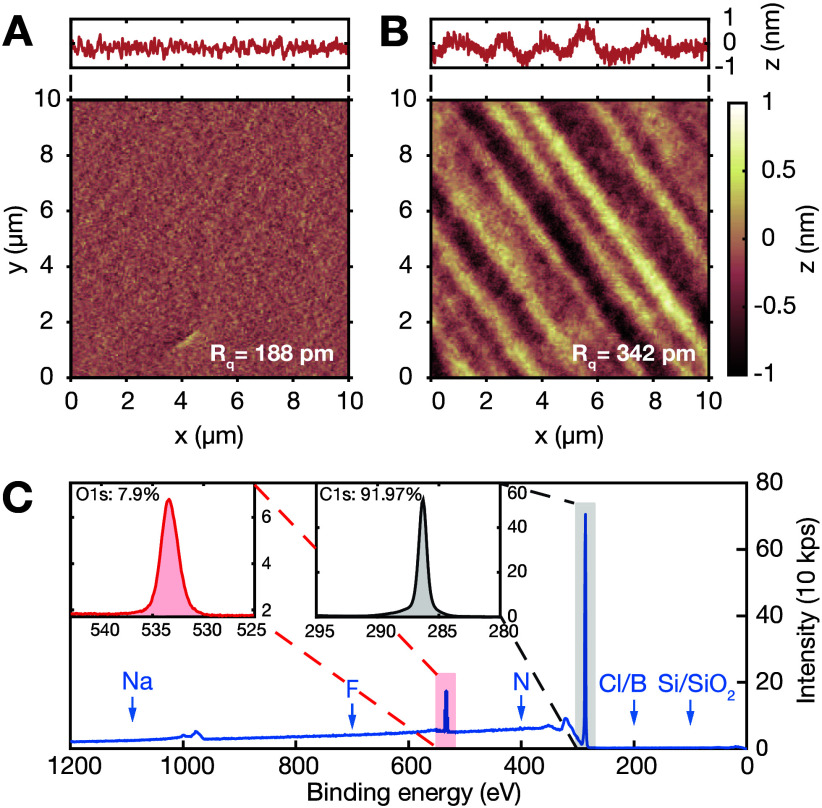
(A) AFM scan of the stress-relieved front diamond surface over an area of 10 × 10 μm^2^. The measured RMS roughness (R_q_) is 188 pm. No increase of R_q_ is observed with the size of the AFM scan (see Supporting Information (SI) Figure S1D). (B) AFM of the deep-etched diamond surface of a ∼900 nm thin platelet over an area of 10 × 10 μm^2^. The R_q_ is 342 pm, while for an area of 1 × 1 μm^2^ is 198 pm. The higher surface roughness is due to different polishing performed on the two surfaces (see SI Figure S1E). (c) XPS survey measurement of the deep-etched surface of a diamond membrane after the LDE. Inset: High-resolution scans of the C 1s and O 1s peaks. The diamond has no contamination and is oxygen terminated after the triacid cleaning with approximately a monolayer of coverage.

The deep-etched back surface was further analyzed by using X-ray photoelectron spectroscopy (XPS) with a beam size of 250 μm^2^ and compared to the front surface. The corresponding XPS survey shown in Figure 3C is identical to that of a stress-relieved surface, exhibiting no detectable contamination. The high-resolution scan of the O 1s peak confirms oxygen termination, with a coverage of approximately one monolayer, consistent with the expected outcome of the triacid cleaning process (refluxing mixture of concentrated perchloric, nitric, and sulfuric acids).^42^ Additionally, the high-resolution scan of the C 1s peak shows no detectable low-energy shoulder, indicating a minimal presence of sp^2^ carbon bonds. These bonds are known to lead to deep traps that, in turn, induce surface magnetic and charge noise, which can degrade the spin and optical coherence of shallow optically active spin defects in diamond.^42^ The absence of contaminants and the low content of sp^2^ carbon-related deep traps confirm the state-of-the-art surface quality achieved by the LDE process.

To assess the relevance of our fabrication approach for quantum photonic devices hosting spin defects, we quantified the charge-noise environment in the fabricated structures, the dominant optical decoherence source for emitters near etched surfaces. This surface noise has motivated alternative approaches that integrate heterogeneous photonic structures with bulk diamond.^68–71^ Using NV centers as sensitive electrometers, we measure the NV centers’ extrinsic optical linewidth in submicrometer-thick platelets and infer an rms electric-field noise of ∼7.4 kV m^–1^ (see SI Sec. V), comparable to state-of-the-art results.^72–74^ For centrosymmetric defects such as the tin-vacancy center, this noise level would broaden the lifetime-limited linewidth by at most 1% (see SI Sec. V), having therefore a negligible impact on their optical coherence.

Next, we focus on the optical lithography process we developed to efficiently define the front pattern of the diamond microstructures. This method relies on focusing the laser of a direct laser writing (DLW) system onto the photoresist, thereby writing the desired pattern, and transferring the pattern to a SiO_2_ hard mask that ensures chemical resistance to O_2_ plasma etching, thereby maintaining high pattern fidelity throughout the process. The fabrication process is illustrated in Figure 4A. We begin with the PECVD deposition of a 300 nm SiO_2_ etch mask and a 60 nm Si layer to facilitate laser focusing. Due to the transparency of both the diamond plate and the SiO_2_ layer, the Si layer is essential to provide the optical contrast needed to focus reliably the feedback laser in the DLW system on the top surface. The DLW optical lithography is performed using a positive photoresist (see SI Sec. IIA), and the pattern is transferred to the SiO_2_/Si layers via a CF_4_ plasma ICP-RIE etch. To prevent contamination and micromasking of the diamond surface, the etch is calibrated so that it stops a few tens of nanometers before reaching the diamond, allowing for photoresist removal. The remaining SiO_2_ and Si focusing layers are then fully removed by a final CF_4_ etch, completing the definition of the etch mask. The pattern is subsequently transferred into the diamond using an O_2_ and CF_4_ plasma ICP-RIE etch, after which the mask is removed via a BOE etch. AFM and XPS measurements confirm that this process does not introduce any contamination or increase the surface roughness.

**Figure 4:**
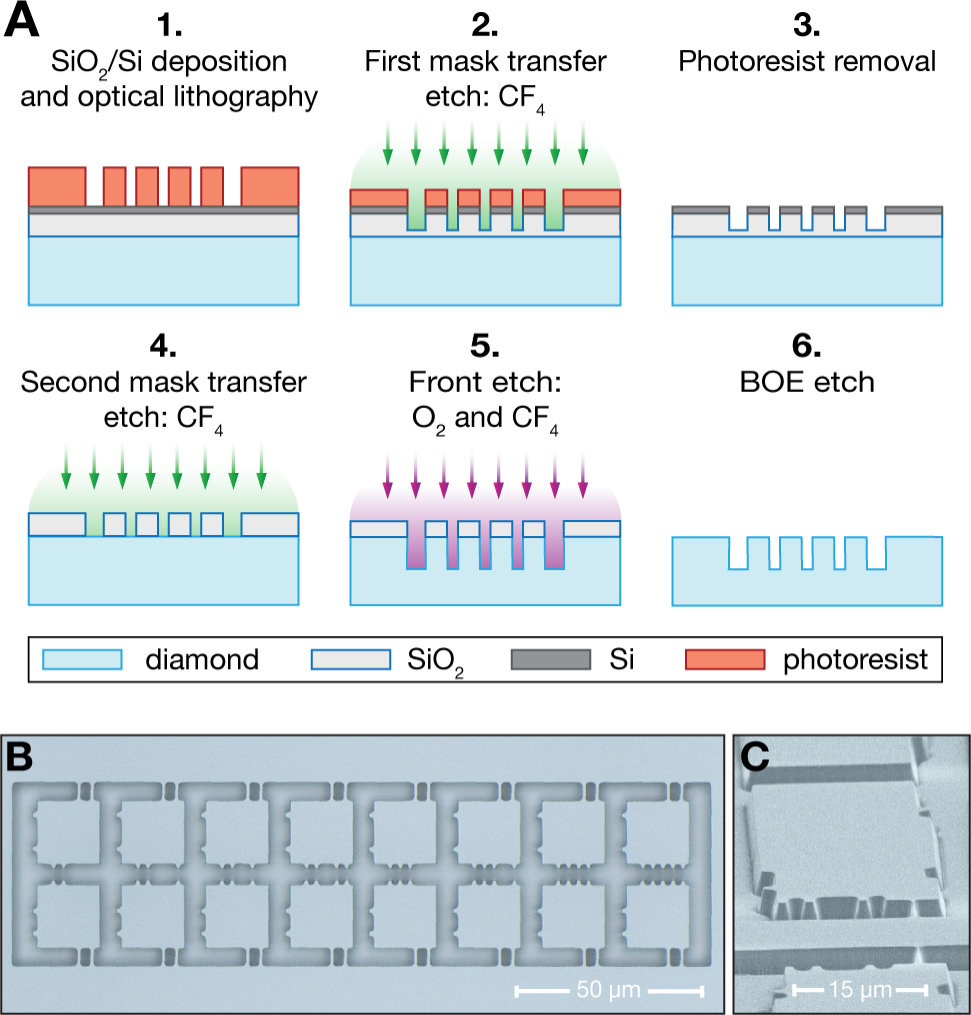
(A) Fabrication flow for the DLW optical lithography and front etching of diamond microstructures. (1) PECVD deposition of the etch mask (300 nm of SiO_2_) and of a 60 nm Si layer for focusing the writing laser. After spin coating the photoresist, DLW optical lithography is performed. (2) Pattern transfer to the SiO_2_/Si layers through a CF_4_ ICP-RIE etch. The etch is calibrated to stop a few tens of nm before etching through the SiO_2_ layer to avoid contamination and micromasking of the diamond surface. (3) Photoresist removal through acetone and piranha cleaning. (4) The remaining few tens of nm of SiO_2_ and the Si layer are removed with CF_4_. (5) Pattern transfer to the diamond front surface via O_2_ and CF_4_ etches. (6) SiO_2_ removal with a BOE etch. (B) Micrograph after pattern definition of 20 × 20 μm^2^ platelets with 1 μm wide bridges via optical lithography. (C) SEM micrograph after pattern definition of 20 × 40 μm^2^ cantilevers with 200 nm wide bridges via optical lithography.

To evaluate our fabrication process, we pattern a 4 × 4 mm^2^ area of the diamond surface with 20 × 20 μm^2^ platelets (see SI Figure S2), each connected to the holding structure by 1 μm-wide bridges, compatible with pick-and-place transfer into a Fabry–Pérot optical microcavity.^27^ A portion of the patterned area is shown in Figure 4B. Additionally, to assess the minimum feature size achievable using positive-tone lithography, we fabricate cantilevers for scanning NV magnetometry,^22^ achieving bridge widths as small as 200 nm (Figure 4C). By leveraging optical lithography with a positive-tone resist and a robust hard-mask transfer technique, we overcome the major limitations of conventional EBL-based approaches, enabling submicrometer resolution over millimeter-scale areas with significantly reduced processing time.

In conclusion, we have demonstrated the fabrication of large-scale, homogeneous, submicrometer thick, free-standing nano- and microstructures from single-crystal bulk diamond, tailored for integrated quantum technologies. A key challenge in deep-etching processes, trench build-up caused by plasma confinement near the mask and diamond etching pit sidewalls, was systematically investigated and mitigated through a lithographic SiO_2_ mask. As a result, we achieved lithographically defined millimeter-scale membranes with thickness gradients as low as 0.35 nm/μm, limited only by the initial wedge of the diamond plate after polishing. This enables the deterministic release of highly uniform nano- and microstructures with thicknesses down to 70 nm. Comprehensive characterization confirms the exceptional quality of the fabricated structures, with atomically smooth, contamination-free surfaces (R_q_ < 200 pm) and a low level of charge noise (∼7.4 kV, rms), ideal for hosting coherent spin defects. Additionally, the resulting nano- and microstructures are compatible with pick-and-place transfer techniques, facilitating integration with heterogeneous material platforms.

To further advance diamond microfabrication, we developed a refined patterning approach based on DLW optical lithography, significantly reducing writing times, simplifying fabrication equipment, and enabling scalable, high-resolution structuring over large areas, making it a promising candidate for large-scale quantum device fabrication. These advancements mark a significant step toward scalable and high-performance diamond-based quantum technologies.

## Supplementary Material



## Notes

Detailed description of the fabrication process: sample preparation and stress relief etch with AFM characterization, front pattern definition, lithographic deep etch with thickness gradient characterization, and thinnest platelet measurement. Description of the platelet breakout process for AFM characterization. Characterization and discussion of optical properties of color centers in submicrometer platelets. (PDF)
