# Supplementary material for: Homogeneous Free-Standing Nanostructures from Bulk Diamond over Millimeter Scales for Quantum Technologies
Source: Nano Lett. 2025 Sep 26;25(40):14526–33. doi: 10.1021/acs.nanolett.5c03083 (PMC12512176; doi:10.1021/acs.nanolett.5c03083)
Supplement: Supplementary file 1 [file nl5c03083_si_001.pdf]

Supporting Information for:
Homogeneous Free-Standing Nanostructures from Bulk Diamond over Millimeter Scales for Quantum Technologies

Andrea Corazza,[1] Silvia Ruffieux,[1] Yuchun Zhu,[2] Claudio A. Jaramillo Concha,[2]
Yannik Fontana,[1] Christophe Galland,[2] Richard J. Warburton,[1] and Patrick Maletinsky[1,*]

[1]*Department of Physics, University of Basel, CH-4056 Basel, Switzerland*
[2]*Institute of Physics and Center for Quantum Science and Engineering, Ecole Polytechnique Fédérale de Lausanne (EPFL), 1015 Lausanne, Switzerland*
(Dated: August 20, 2025)

In this supporting information, we provide details for the developed fabrication processes, which can be broken down into three main steps, as well as descriptions of the performed characterizations:

I. Sample preparation and stress relief etch (SRE)

II. Front pattern definition

III. Lithographic deep etch (LDE)

IV. Platelet breakout for AFM characterization

V. Optical properties of color centers in sub-micron platelets

Unless specified, all dry etch processes are done using inductively-coupled reactive ion etching (ICP-RIE) in a system from Sentech (SI 500), with the diamonds glued on silicon chips with Crystalbond 509. Etch parameters are listed in Table I. Wet etching is performed either in a buffered oxide etchant (BOE) 10:1, in a piranha solution (sulfuric acid and hydrogen peroxide, 3:1) or in a refluxing mixture of concentrated perchloric, nitric, and sulfuric acids (triacid, 1:1:1). To ensure that the processes do not lead to contamination or morphological damage, surface quality is assessed at regular intervals using X-ray photoelectron spectroscopy (XPS) and atomic force microscopy (AFM), following Ref. [1].

TABLE I. ICP-RIE plasma parameters for the SRE, front pattern definition, and the LDE. The mask transfer and front etch are carried out using a (6-inch) silicon carrier inside the reactor, while the SRE and LDE, which involves Ar/Cl_2 plasma, were carried out using a ceramics carrier wafer.

Plasma	ICP power (W)	RF power/bias (W)	Flux (sccm)	Pressure (Pa)	Etch rate (nm/min)
Ar/Cl_2 [a]	400	100	25/40	1	diamond: 40, SiO_2: 90
O_2 [a]	700	50	60	1.3	diamond: 180, SiO_2: 2
CF_4 [b]	50	45	30	0.4	Si: 50, SiO_2: 40, photoresist: 60
O_2 [c]	500	100	50	0.5	diamond: 160
O_2 [d]	400	200	30	1.3	diamond: 60

[a] SRE and LDE
[b] Mask transfer etch and front etch – optical lithography
[c] Front etch – optical lithography + sample E
[d] Front etch – e-beam lithography sample C

I. SAMPLE PREPARATION AND STRESS RELIEF ETCH

We fabricate our samples from commercially available synthetic single-crystal diamond plates grown by chemical vapor deposition (CVD). Sample A is a 50 µm-thick optical grade (100) diamond plate, double-side polished to R_q <3 nm ($[N_S]$ < 1 ppm, [B] < 5 ppb, Delaware Diamond Knives). Samples B, C, D and E are electronic grade (100) plates ($[N_S]$ < 5 ppb, [B] < 1 ppb, Element Six), that were laser-diced into 50 µm-thick membranes (Almax easyLab). Samples B and C are polished on the front

* patrick.maletinsky@unibas.ch

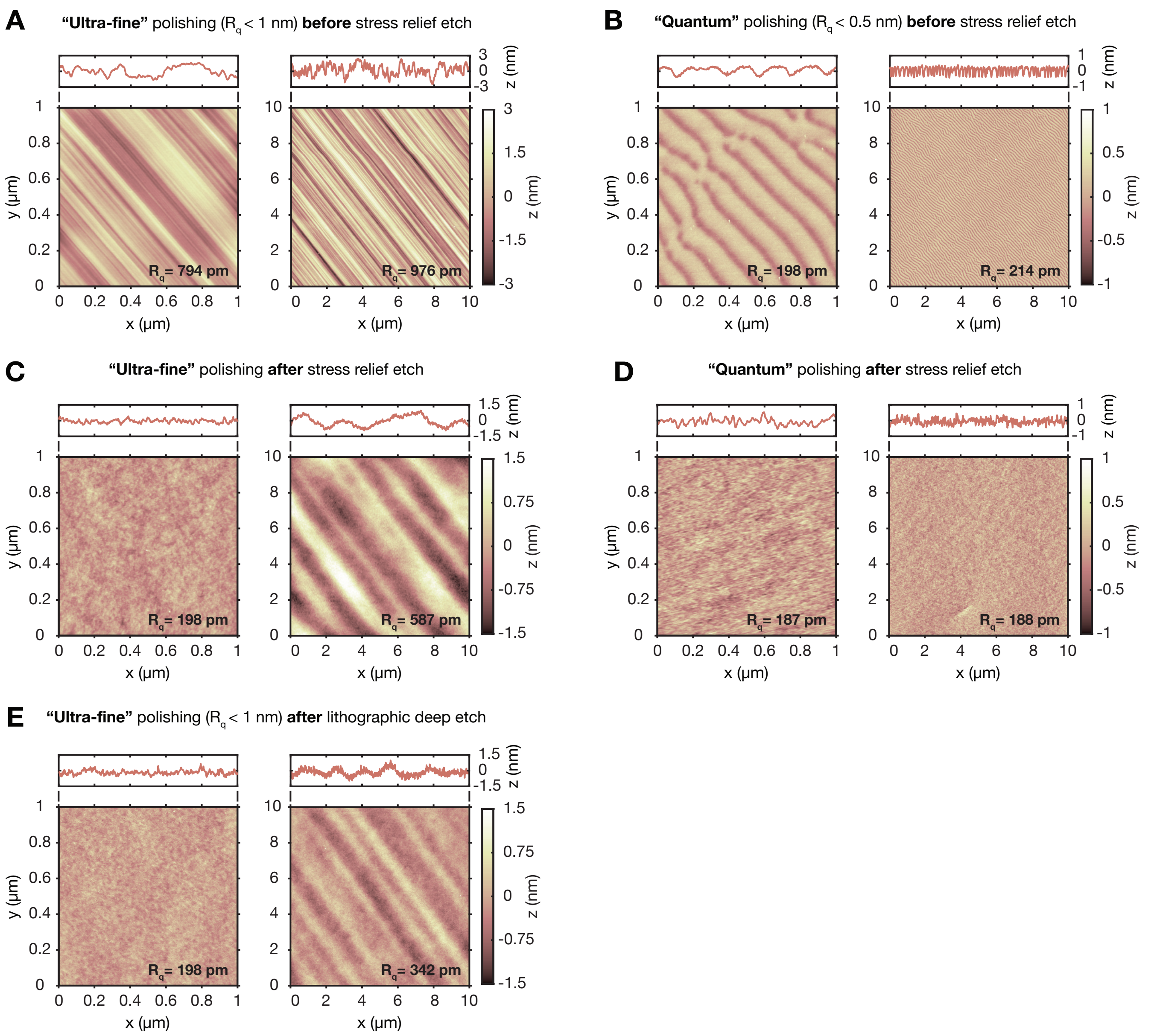

FIG. S1. $1 \times 1\,\mu m^2$ and $10 \times 10\,\mu m^2$ AFM scans of the diamond surface for the two different polishing methods "Ultra-Fine Polishing" and "Quantum Polishing" performed by Almax easyLab at different processing steps.

side to R_q <0.5 nm ("Quantum polishing", Almax easyLab) and on the back to R_q <1 nm ("Ultra-fine polishing", Almax easyLab). Sample D is double-sided polished to R_q <1 nm ("Ultra-fine polishing", Almax easyLab), while sample E is double-sided polished to R_q <0.5 nm ("Quantum polishing", Almax easyLab). Before any process, the samples were cleaned in triacid. The AFM characterization reveals that the "Ultra-fine polishing" method achieves a peak-to-peak waviness of 6 nm and a typical surface roughness of $R_q = 794$ pm over an area of $1 \times 1\,\mu m^2$, which further increases to $R_q = 976$ pm over an area of $10 \times 10\,\mu m^2$ (Fig. S1 A). In contrast, the "Quantum polishing" method exhibits a dramatically reduced peak-to-peak waviness of 1.5 nm, and the surface roughness remains nearly constant when the analyzed area increases from $1 \times 1\,\mu m^2$ ($R_q = 198$ pm) to $10 \times 10\,\mu m^2$, with a value as low as $R_q = 214$ pm (Fig. S1 B). The low waviness and exceptional smoothness achieved through "Quantum polishing" hold significant promise for fabricating diamond surfaces that can increase the performances of diamond photonic devices [2]. The 50 µm-thick diamond plates used to realize the free-standing structures exhibit a wedge between 0.25 µm and 2.5 µm across the 4.5 mm lateral dimension after laser slicing and polishing. As an example, sample C exhibits wedges across the 4 sides ranging between 0.25 and 1.4 µm.

Sub-surface crystal damage and strain induced by mechanical polishing can extend micrometers-deep [3–5], and is extremely detrimental to the color centers embedded in the material [1, 6, 7]. A damage-free lattice is recovered by etching away ∼4 µm

of diamond from the front surface using Ar/Cl_2 and O_2 plasma. Starting this stress relief etch (SRE) with an extended Ar/Cl_2 plasma step is crucial: chlorine chemistry removes the damaged layer without propagating lattice defects into the crystal [4]. A last O_2 step removes possible deleterious Cl_2 contamination [8]. The recipe used for all samples was 8 cycles of (300 s Ar/Cl_2 + 150 s O_2) with the plasma parameters specified in Table I. The AFM characterization of the "ultra-fine" polished surface shows significantly reduced surface waviness and roughness after the SRE, with the peak-to-peak waviness decreasing by a factor of four to 1.5 nm and a roughness (R_q) of 198 pm on a $1 \times 1\,\mu m^2$ area, and by nearly a factor of two to 3 nm and R_q of 587 pm on a $10 \times 10\,\mu m^2$ area (Fig. S1 C). The smoothing effect of the etching process is less pronounced on the "quantum" polished surface, where the surface roughness slightly decreases to R_q = 188 pm and remains constant up to an area of $10 \times 10\,\mu m^2$, while the waviness becomes more randomized (Fig. S1 D). Although the differences in surface waviness and roughness between the two polishing processes appear negligible over a $1\,\mu m^2$ area, the improvements in morphology and roughness are significant over larger areas spanning several μm^2.
An XPS characterization is performed to check for the most common diamond contaminants (B, F, Na, Si/SiO_2, Cl). An XPS survey spectrum after the stress relief etch is shown in Fig. 3 C of the main text, showing no detectable amounts of contaminants. The detected oxygen is the one terminating the diamond surface after the triacid cleaning.

II. FRONT PATTERN DEFINITION

In this work, we fabricate two main types of free-standing structures: diamond platelets – used either to functionalize Fabry-Pérot microcavities or as cantilevers for scanning NV magnetometry (samples A, B, D and E) – and photonic crystal (PhC) cavities and waveguides with a distributed Bragg reflector (DBR) designed for the singlet transition at 1042 nm of the NV center (sample C). The first type consists of $20\text{-}40 \times 20\,\mu m^2$ diamond platelets, attached to a holding bar by 5 µm long and 0.2-1 µm wide bridges. The second type, PhC cavities and waveguides, have a width of 535–635 nm, a DBR periodicity of 265–300 nm, and a tapered section ending at a thickness of 50 nm. For the fabrication of PhC cavities and waveguides, we employ a well-established process based on electron beam lithography (EBL) using FOx-16 (DuPont, formerly Dow Corning) as a resist. In contrast, for the fabrication of diamond platelets, we develop a refined process based on optical lithography.

A. Optical lithography: samples A, B and D

The hard mask used for the front pattern definition via optical lithography consists of a 300 nm SiO_2 layer deposited through plasma-enhanced chemical vapor deposition (PECVD) at 300 °C (Oxford Instruments, PlasmaPro 80 PECVD) on the diamond's front surface. Optical lithography is performed with a direct laser writer (DLW) equipped with an all-optical auto-focus module (Heidelberg Instruments, uPG 101). Since the diamond plate and the SiO_2 layer do not have enough optical contrast to guarantee reliable focusing of the all-optical auto-focus module on the top surface, a 60 nm Si layer was deposited via PECVD at 300 °C on top of the SiO_2 hard mask without breaking vacuum. AR 300-80 New (Allresist GmbH) is applied as an adhesion promoter

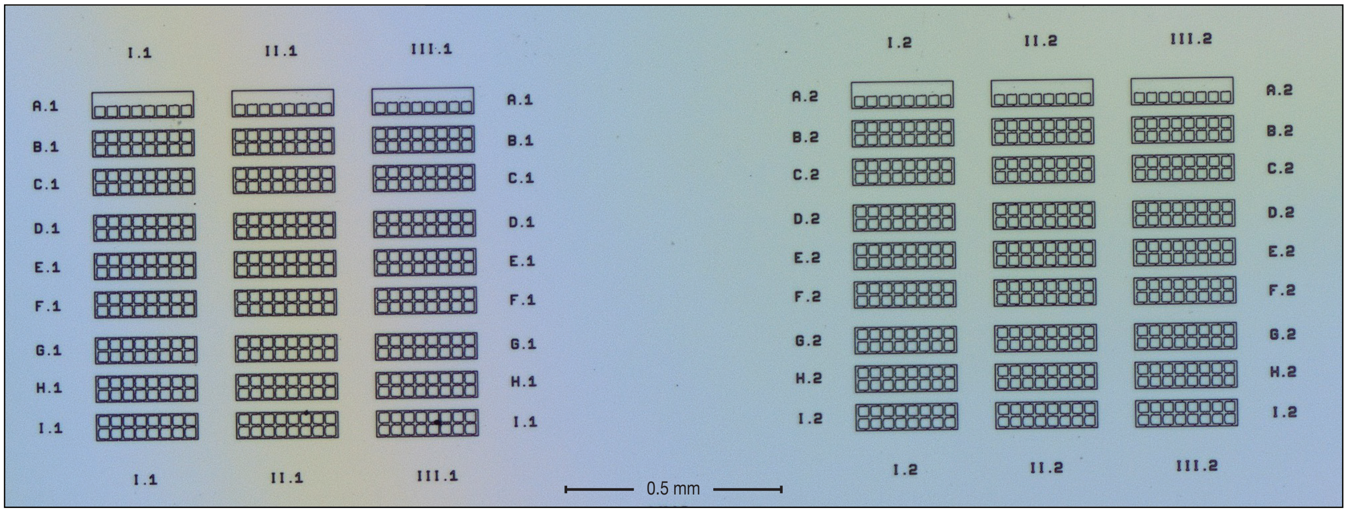

FIG. S2. Micrograph of sample B after front pattern definition of two 1 mm^2 arrays of $20 \times 20\,\mu m^2$ platelets with 1 µm wide bridges by optical lithography.

and S1813 (Shipley, Microposit) was chosen as photoresist, forming a 1.3 μm thick layer. The pattern is written using the all-optical auto-focus function of the DLW at 7.5 mW power, and developed with MF-319 (Shipley, Microposit). The pattern is then transferred from the photoresist to the Si/SiO_2 mask using ICP-RIE with a CF_4 plasma. The etching parameters are listed in Table I. This process removes the Si layer and approximately 260 nm of the SiO_2 mask, allowing photoresist removal (with acetone and a piranha clean) without contaminating the diamond surface. To complete the mask transfer etch, an additional CF_4 etch is performed to remove the remaining Si layer and the last ~40 nm of the hard mask. The pattern is then transferred from the SiO_2 hard mask to the diamond using an O_2 and CF_4 ICP-RIE process that etches approximately 3 μm of diamond material and effectively transfers the desired microstructure. The recipe used is 8 cycles of (10s CF_4 + 120s O_2). The detailed etching parameters for this process are provided in Table I. The CF_4 plasma plays a crucial role in preventing micromasking: the etch rate of Si, SiO_2, and photoresist in CF_4 plasma is much higher than that of diamond. Using a combined CF_4 and O_2 plasma for the diamond etch removes redeposited SiO_2 on the etched diamond surface, leading to smooth etched surfaces [9]. The hard mask is finally removed with a BOE etch and the sample is cleaned using a triacid clean. In Fig. S2 a micrograph of two 1 mm^2 arrays of diamond platelets patterned on sample B is shown. AFM and XPS characterizations were performed to verify that none of the fabrication steps damaged the diamond surface.

B. Electron beam lithography: samples C and E

For front pattern definition via electron beam lithography, we use FOx-16 both as a resist and a hard mask, a common approach for fabricating diamond micro- and nanostructures [10]. To prevent charge buildup during writing, for sample E Electra 92 (Allresist) is spin coated on top of FOx-16 and for sample C a 20 nm Ti layer was e-beam evaporated (Alliance-Concept EVA 760) onto the front surface of the diamond before spin coating the resist. The Ti layer also improves adhesion between the resist and the diamond surface, which is more important for small nanostructures. The FOx-16 layer thickness ranges between 450 nm (sample E) and 1.25 µm (sample C) depending on the spinning conditions, and the pattern was written using either a 30 KeV (sample E) or a 100 keV (sample C) electron beam system (Zeiss Supra with Raith Elphy module or Raith EBPG5000, respectively). The resist was then developed in tetramethylammonium hydroxide (TMAH). The hard mask design was then transferred to the diamond surface using an O_2 ICP-RIE etch, etching 3.5 µm (sample E) and 1.25 µm (sample C) of diamond material (Sentech SI 500 and Oxford Instruments PlasmaPro100 Cobra, respectively). The parameters used are reported in Table I. Finally, the FOx-16 mask was removed with a BOE etch, and the sample was cleaned using a triacid cleaning process.

III. LITHOGRAPHIC DEEP ETCH (LDE)

The lithographic deep etch process begins by protecting the front surface of the diamond with a 50-100 nm SiO_2 layer, deposited via PECVD at 300 °C. The sample is then flipped and a 10-22 µm SiO_2 layer is deposited on the backside. Next, we define the etching windows of the hard mask by optical lithography using the same DLW system as for the front lithography. AR 300-80 New is applied as adhesion promoter and AZ 4562 (MicroChemicals GmbH) as photoresist, resulting in a ~5 µm thick layer that offers high etch resistance to BOE. For patterning the deep etch windows, we align the design to the front pattern that is visible through the mask and the diamond plate. As submicrometer resolution is not required, we manually set the writing distance and omit the Si focusing layer, which is used for the all-optical autofocus system. We use a laser power of 120 mW and develop the resist in AZ 400K 1:4 (MicroChemicals GmbH). The lithographic design is then transferred to the SiO_2 hard mask via wet etching in BOE with an etching rate of 200 nm/min. We then remove the photoresist using acetone and a piranha clean.

The isotropic wet etch of the lithographic hard mask produces a non-uniform sidewall angle, considerably less than 45° near the diamond surface. Combined with the selection of plasma gases for deep etching – which gradually erodes the mask, causing it to retract concurrently with the diamond etch – this phenomenon forms the basis of the process's key principle: reducing plasma confinement and, consequently, the ion flux near both the mask and the diamond etch pit sidewalls. It is important to note that over-etching the deep etch mask creates steeper sidewall angles adjacent to the diamond surface, leading to the formation of a shallow trench that may eventually perforate the diamond membrane (see Fig. S3).

Although CF_4 and SF_6 are effective in etching SiO_2, we opted for Ar/Cl_2, motivated by the excellent surface quality obtained after the SRE. Ar/Cl_2 also exhibits greater chemical selectivity for SiO_2 over diamond. O_2 plasma, on the other hand, shows a higher etch rate for diamond compared to SiO_2. By employing the same plasma chemistry and parameters as in the SRE process (see Table I) – Ar/Cl_2 and O_2 gases – we create diamond terraces via preferential etching of either diamond or SiO_2 by switching between the two plasmas. This allows us to control the angle of the diamond etching pit sidewall by adjusting the Ar/Cl_2:O_2 ratio. To investigate the process, we used two different etching recipes:

- sample A: 150 s Ar/Cl_2 + (90 s Ar/Cl_2 + 300 s O_2)×N – Ar/Cl_2:O_2 ratio = 0.3
- samples B, C: 150 s Ar/Cl_2 + (150 s Ar/Cl_2 + 150 s O_2)×N – Ar/Cl_2:O_2 ratio = 1

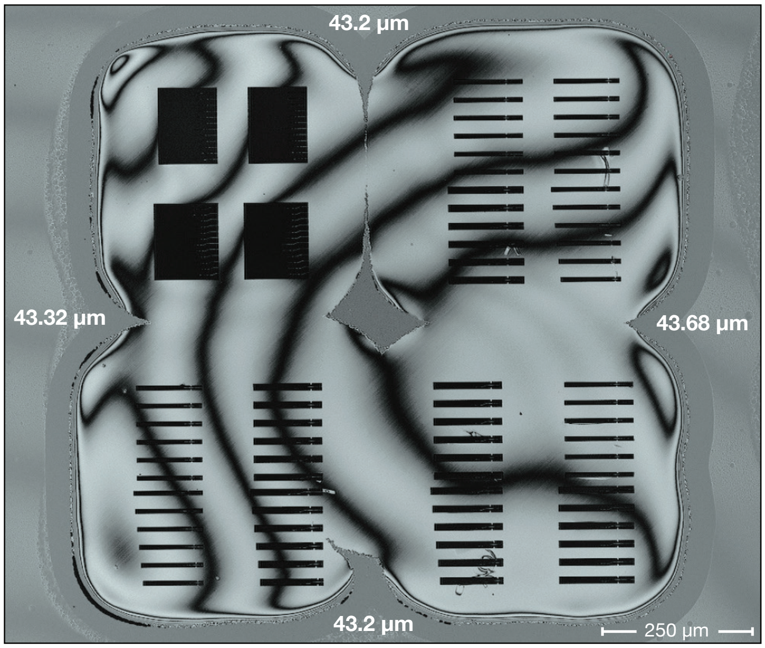

FIG. S3. Laser scanning confocal microscope image (Keyence VK-X1100) showing a $1 \times 1\,\text{mm}^2$ section of a free-standing membrane patterned with PhC and waveguides. The membrane exhibits a wedge of 0.35 nm/μm in the direction of the highest thickness gradient, with a thickness difference of 84 nm between fringes set by the laser wavelength $\lambda = 404\,\text{nm}$. This value matches the wedge of the diamond plate measured on the 4 sides of the writefield via interferometric measurements with the same confocal microscope.

where N is the number of cycles needed to release the structures with the desired thickness. Similarly to the SRE, the deep etch always ends with an O_2 plasma step. Following the etch, the lithographic mask and front-side protection were removed through a BOE etch, and any residual contamination was eliminated by a triacid clean. AFM and XPS characterizations were carried out to confirm that none of the fabrication steps damaged or contaminated the diamond surface, and the results are shown in Fig. S1 E and in Fig. 3 C of the main text. As visible from the peak-to-peak waviness and R_q values of the $10 \times 10\,\mu\text{m}^2$ scans shown in Fig. S1 C and S1 E, the LDE smoothens even more the polishing-induced waviness than the SRE thanks to the longer exposure to Ar/Cl_2 plasma.

The thickness gradient of the diamond membranes after the LDE was also characterized. To understand whether our deep etch strategy introduces further thickness inhomogeneities in the fabricated free-standing membranes, we measured the thickness of the diamond plate via interferometric measurements using a confocal microscope (Keyence VK-X1100, $\lambda = 404\,\text{nm}$) around the writefields patterned with the micro- and nanostructures before the LDE process. We then compared the thickness difference across the 4 sides of the writefields, with the one that can be extracted from a laser scanning confocal image performed with the same microscope, as shown in Fig. S3. Knowing that the thickness difference between fringes is 84 nm and counting the number of fringes, one can compare the thickness gradient of the patterned area before and after the deep etch. We show that the LDE process does not introduce further thickness inhomogeneities to the starting wedge of the diamond plate caused by laser slicing and polishing and the membrane exhibits a wedge of 0.35 nm/μm in the direction of the highest thickness gradient.

Fig. S4 shows a 70 nm thick platelet fabricated with the LDE. It is coated with 10 nm of Ti for efficient discharging during the SEM investigation and measured at a viewing angle of 70°. The height measurement in Fig. S4 does not take into account the viewing angle, and so the real height is 6% larger than the measured one.

It has to be noted that while our deep etch fabrication flow can be applied to a large range of starting thicknesses of the diamond plate, for free-standing structures with sub-micrometer thickness we found that 50 μm is a good compromise between mechanical stability, processing times, and thickness of the SiO_2 hard mask needed to release the structures with no trench along the etching pit sidewall.

Lastly, we have found that the LDE is also a powerful tool when it comes to releasing several micrometer-thick structures such as cantilevers for scanning NV magnetometry, as it allows us to deterministically release all devices in large arrays. Depending on the thickness of the structures to be released and the initial wedge of the diamond, trench formation is less of an issue, meaning that the LDE can be sped up using a lower Ar/Cl_2:O_2 ratio such as 0.3, which also significantly reduces the required thickness of the SiO_2 hard mask.

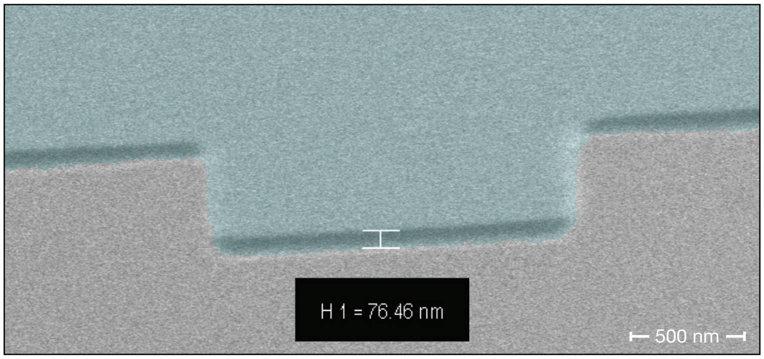

FIG. S4. SEM micrograph at 70° viewing angle of a 70 nm thick platelet coated with 10 nm of Ti as a discharging layer.

IV. PLATELET BREAKOUT FOR AFM CHARACTERIZATION

To characterize the LDE back surface with AFM, we breakout some free-standing diamond platelets onto a Si chip using a home-assembled micromanipulation station. Glass microprobes, crafted with a glass needle puller, are utilized to snap the bridge connecting the platelet to the holding bar by applying pressure to the platelet's surface, as shown in Fig. S5. The surface orientation of the platelet that lands on the silicon chip is determined using non-mirror-symmetric binary code markers added to the platelet (see Fig. S5). Once the back surface is correctly oriented facing upwards, we gently maneuver the platelet with the microprobes until van der Waals forces bond it to the Si chip, and it therefore cannot be moved anymore.

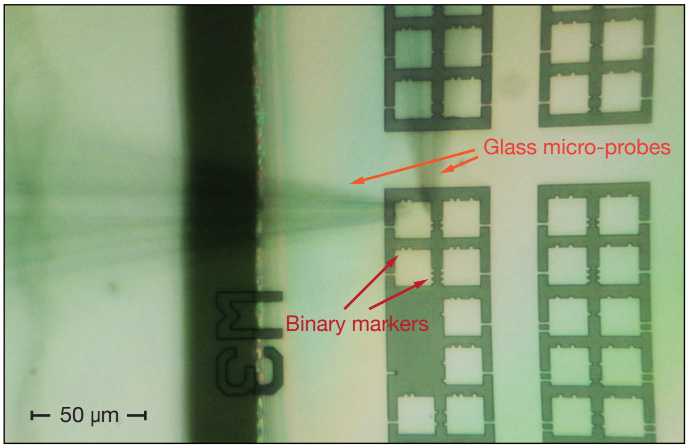

FIG. S5. Platelet deposition with a micromanipulation station using glass micro-probes. The specific platelet is identified via binary markers that helps identifying also the front from the back surface.

V. OPTICAL PROPERTIES OF COLOR CENTERS IN SUB-MICRON PLATELETS

Free-standing diamond nanostructures hosting optically-active single spin qubits are of particular interest for quantum photonics applications. Top-down fabrication techniques often introduce charge noise, compromising optical linewidths. We show that our fabrication process preserves a low-noise environment, standing comparison with other state-of-the-art approaches.

A. Impact of LDE on the optical linewidth of nitrogen vacancy centers

We probe the local charge noise level by measuring the extrinsic broadening of nitrogen-vacancy (NV) centers' optical transitions. The C_{3v} symmetry of NV centers leads to a large permanent dipole moment and thus a high sensitivity to electrostatic fluctuations.

The sample containing NV centers is sample E. The front patterning of arrays of $20 \times 20\,\mu m^2$ platelets is performed by EBL, as described in Sec. II B, and the release is performed with the LDE approach outlined in the main text until the membrane reaches a thickness of 8 µm. A final step using a quartz mask leads to micro-platelets with thicknesses in the range 0.5 µm to 3.5 µm. The change in masking method is purely functional: it deliberately introduces a thickness gradient. After fabrication and prior to implantation, the sample undergoes a multi-step annealing process to remove subsurface damage caused by ICP etching. Annealing was conducted in a home-built high-vacuum oven at a base pressure below 10^{-6} mbar and the process is adapted from Ref. [1]: 5 hours at 100 °C, 8 hours at 400 °C, 8 hours at 800 °C, and 2 hours at 1200 °C, with 6-hour ramp times between steps. After the annealing the sample is implanted with $^{12}C^{+}$ ions following the implantation post-fabrication concept presented in Ref. [6]. Ions are implanted at two different energies: 50 keV (2.75×10^8 ions/cm^2) and 110 keV (4.5×10^8 ions/cm^2). The dual implantation is specific to the end-use of the sample and has no known consequences on the results discussed here. The sample is again annealed using the same process as the prior-implantation annealing. Finally, the sample is subjected to oxygen annealing following the recipe described in Ref. [1]. Through an AFM characterization performed after the oxygen anneal, illustrated in Fig. S6 with panels (A) and (B) displaying $1 \times 1\,\mu m^2$ and $10 \times 10\,\mu m^2$ scan areas, respectively, we notice that the surface roughness increases after the process, from $R_q < 0.2$ nm to $R_q \sim 0.7$ nm, indicating partial graphitization during the annealing. For comparison, the AFM scans showing the same surface morphology and roughness of both the front and bottom sides of the platelets prior to annealing are shown in Fig. S1 D. Tuning more carefully the oxygen annealing temperature would avoid the increase in surface roughness due to graphitization while maintaining the benefits due to the surface termination.

The extrinsic optical linewidths of single NV centers are measured in the same setup and following the methodology detailed in Ref. [11]. The linewidth measurement sequence is shown in Fig. S7 A. Prior to each weak resonant probe (637 nm, 5 nW), the NV center is repumped by a green laser (532 nm, 220 µW) in order to reset its charge and spin state. A side effect of the repump pulse is the shuffling of charges localized on traps within and at the surfaces of the diamond matrix. The trap density and proximity to the NV center influence the amount of extrinsic broadening experienced by the emitter (Fig. S7 B). Fig. S7 C shows an NV center presenting an extrinsically broadened linewidth of ∼225 MHz. This value is comparable to extrinsically broadened linewidths measured elsewhere on similar (albeit at least twice thicker) diamond membranes [6, 12, 13]. The linewidth measurement allows us to derive an extrinsic rms electric-field noise noise. Taking a consensual value for the NV center permanent dipole moment

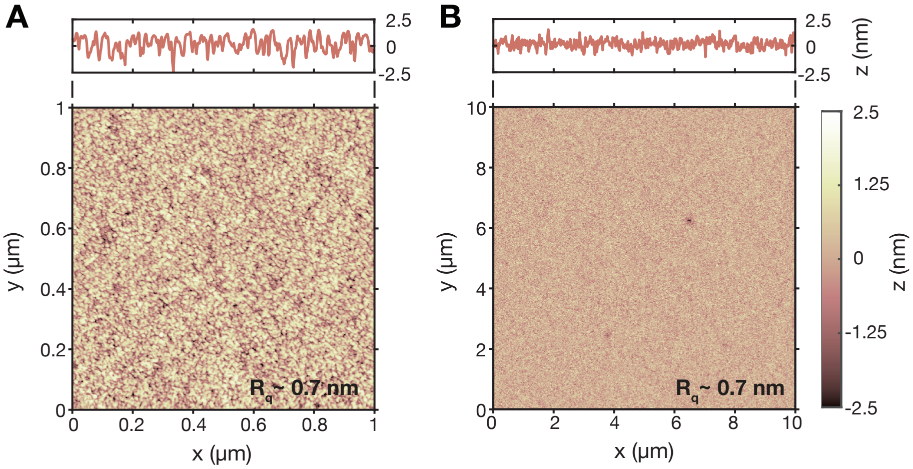

FIG. S6. AFM scans of the sample following oxygen annealing: (A) $1 \times 1\,\mu m^2$ scan area, and (B) $10 \times 10\,\mu m^2$ scan area. Both the front and back surfaces of the free-standing membrane retain the large-scale flatness observed prior to annealing (see Fig. 3), while the surface roughness increases from $R_q < 0.2$ nm to $R_q \sim 0.7$ nm.

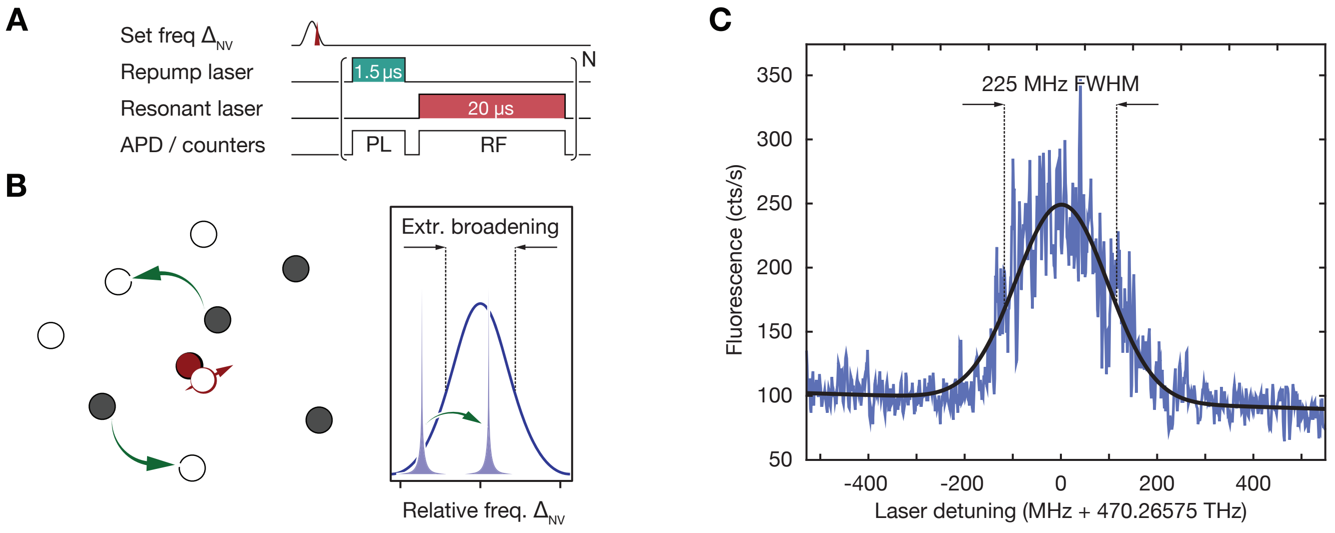

FIG. S7. Charge noise sensed with a single NV center. (A) Pulse sequence interleaving charge-noise-inducing repump pulses and readout pulses. (B) Besides re-initializing the NV center charge and spin state, the 532 nm repump pulse also triggers spatial reconfigurations of elementary charges (green arrows) in the sample. This results in a varying center frequency of the target NV center, materializing in the extrinsic broadening of the measured linewidth. (C) Typical resonance fluorescence spectrum for a single NV center in a ∼800 nm-thick micro-platelet.

(1.35 Debye) and limiting ourselves to first-order effects, we find an rms noise of ∼7.3 kV/m [14, 15].

B. Impact on centrosymmetric emitters

In recent years, emphasis has been put on finding and investigating optically-active spins in diamond displaying reduced sensitivity to charge noise. Silicon-, germanium-, and more recently tin-vacancy (SnV) centers, with their D_{3d} symmetry are first-order insensitive to electric fields. While data for silicon- and germanium-vacancy centers is lacking, De Santis *et al.* measured SnV centers in bulk and reported an average dipole moment of 0.4×10^{-4} Debye and a volume polarizability of 0.23 Å^3 [16]. Aghaeimeibodi *et al.* reported significantly larger average values in nanopillars: 2.9×10^{-3} Debye and 3.4 Å^3 [17]. The discrepancy may be explained by fabrication-induced local symmetry-breaking strain fields.

We can extrapolate the amount of extrinsic broadening in the optical linewidths if such emitters were to be created in our devices. The extra-broadening, calculated up to second-order, ranges from 3.9×10^{-3} MHz (using bulk values) to 2.8×10^{-1} MHz (using nanopillar values). Compared to the SnV's Fourier-transform-limited linewidth (26 MHz), the extra broadening leads at most to a 1% increase. We conclude that the optical coherence of centrosymmetric defects would not be affected by our fabrication technique.

[1] S. Sangtawesin, B. L. Dwyer, S. Srinivasan, J. J. Allred, L. V. Rodgers, K. De Greve, A. Stacey, N. Dontschuk, K. M. O'Donnell, D. Hu, D. A. Evans, C. Jaye, D. A. Fischer, M. L. Markham, D. J. Twitchen, H. Park, M. D. Lukin, and N. P. De Leon, Origins of Diamond Surface Noise Probed by Correlating Single-Spin Measurements with Surface Spectroscopy, Phys. Rev. X **9**, 031052 (2019).

[2] S. Flågan, D. Riedel, A. Javadi, T. Jakubczyk, P. Maletinsky, and R. J. Warburton, A diamond-confined open microcavity featuring a high quality-factor and a small mode-volume, J. Appl. Phys. **131**, 113102 (2022).

[3] P.-N. Volpe, P. Muret, F. Omnes, J. Achard, F. Silva, O. Brinza, and A. Gicquel, Defect analysis and excitons diffusion in undoped homoepitaxial diamond films after polishing and oxygen plasma etching, Diam. Relat. Mater. **18**, 1205 (2009).

[4] I. Friel, S. Clewes, H. Dhillon, N. Perkins, D. Twitchen, and G. Scarsbrook, Control of surface and bulk crystalline quality in single crystal diamond grown by chemical vapour deposition, Diam. Relat. Mater. **18**, 808 (2009).

[5] M. Naamoun, A. Tallaire, F. Silva, J. Achard, P. Doppelt, and A. Gicquel, Etch-pit formation mechanism induced on HPHT and CVD diamond single crystals by H 2/O 2 plasma etching treatment: Part of topical section on fundamentals and applications of diamond, Phys. Status Solidi A **209**, 1715 (2012).

[6] V. Yurgens, A. Corazza, J. A. Zuber, M. Gruet, M. Kasperczyk, B. J. Shields, R. J. Warburton, Y. Fontana, and P. Maletinsky, Spectrally

stable nitrogen-vacancy centers in diamond formed by carbon implantation into thin microstructures, Appl. Phys. Lett. **121**, 234001 (2022).

[7] L. Rondin, J. P. Tetienne, T. Hingant, J. F. Roch, P. Maletinsky, and V. Jacques, Magnetometry with nitrogen-vacancy defects in diamond, Rep. Prog. Phys. **77**, 5 (2014).

[8] Y. Tao, J. M. Boss, B. A. Moores, and C. L. Degen, Single-crystal diamond nanomechanical resonators with quality factors exceeding one million, Nat. Commun. **5**, 3638 (2014).

[9] T. Yamada, H. Yoshikawa, H. Uetsuka, S. Kumaragurubaran, N. Tokuda, and S. ichi Shikata, Cycle of two-step etching process using ICP for diamond MEMS applications, Diam. Relat. Mater. **16**, 996 (2007).

[10] P. Appel, E. Neu, M. Ganzhorn, A. Barfuss, M. Batzer, M. Gratz, A. Tschöpe, and P. Maletinsky, Fabrication of all diamond scanning probes for nanoscale magnetometry, Rev. Sci. Instrum. **87**, 063703 (2016).

[11] V. Yurgens, Y. Fontana, A. Corazza, B. J. Shields, and P. Maletinsky, Cavity-assisted resonance fluorescence from a nitrogen-vacancy center in diamond, npj Quantum Inf. **10**, 112 (2024).

[12] M. Kasperczyk, J. A. Zuber, A. Barfuss, J. Kölbl, V. Yurgens, S. Flågan, T. Jakubczyk, B. Shields, R. J. Warburton, and P. Maletinsky, Statistically modeling optical linewidths of nitrogen vacancy centers in microstructures, Phys. Rev. B **102**, 75312 (2020).

[13] M. Ruf, M. IJspeert, S. Van Dam, N. De Jong, H. Van Den Berg, G. Evers, and R. Hanson, Optically coherent nitrogen-vacancy centers in micrometer-thin etched diamond membranes, Nano Lett. **19**, 3987 (2019).

[14] P. Tamarat, T. Gaebel, J. Rabeau, M. Khan, A. Greentree, H. Wilson, L. Hollenberg, S. Prawer, P. Hemmer, F. Jelezko, *et al.*, Stark shift control of single optical centers in diamond, Phys. Rev. Lett. **97**, 083002 (2006).

[15] T. Delord, R. Monge, G. Lopez-Morales, O. Bach, C. E. Dreyer, J. Flick, and C. A. Meriles, Probing electric-dipole-enabled transitions in the excited state of the nitrogen-vacancy center in diamond, arXiv preprint arXiv:2405.16280 10.48550/arXiv.2405.16280 (2024).

[16] L. De Santis, M. E. Trusheim, K. C. Chen, and D. R. Englund, Investigation of the stark effect on a centrosymmetric quantum emitter in diamond, Phys. Rev. Lett. **127**, 147402 (2021).

[17] S. Aghaeimeibodi, D. Riedel, A. E. Rugar, C. Dory, and J. Vučković, Electrical tuning of tin-vacancy centers in diamond, Phys. Rev. Appl. **15**, 064010 (2021).
